# Apoptosis, autophagy, necroptosis, and cancer metastasis

**DOI:** 10.1186/s12943-015-0321-5

**Published:** 2015-02-21

**Authors:** Zhenyi Su, Zuozhang Yang, Yongqing Xu, Yongbin Chen, Qiang Yu

**Affiliations:** Department of Biochemistry and Molecular Biology, Medical School, Southeast University, Nanjing, Jiangsu 210009 China; Department of Cell Biology, Harvard Medical School, Boston, MA 02115 USA; Bone and Soft Tissue Tumors Research Center of Yunnan Province, Department of Orthopaedics, the Third Affiliated Hospital of Kunming Medical University (Tumor Hospital of Yunnan Province), Kunming, Yunnan 650118, China; Department of Orthopaedics, Kunming General Hospital of Chengdu Military Command, Kunming, Yunnan 650118 China; Key Laboratory of Animal Models and Human Disease Mechanisms, Kunming Institute of Zoology, Chinese Academy of Sciences, Kunming, Yunnan 650223 China; Shanghai Institute of Materia Medica, Chinese Academy of Sciences, 555 Zuchongzhi Road, Shanghai, 201203 China

**Keywords:** Apoptosis, Autophagy, Necroptosis, Metastasis

## Abstract

Metastasis is a crucial hallmark of cancer progression, which involves numerous factors including the degradation of the extracellular matrix (ECM), the epithelial-to-mesenchymal transition (EMT), tumor angiogenesis, the development of an inflammatory tumor microenvironment, and defects in programmed cell death. Programmed cell death, such as apoptosis, autophagy, and necroptosis, plays crucial roles in metastatic processes. Malignant tumor cells must overcome these various forms of cell death to metastasize. This review summarizes the recent advances in the understanding of the mechanisms by which key regulators of apoptosis, autophagy, and necroptosis participate in cancer metastasis and discusses the crosstalk between apoptosis, autophagy, and necroptosis involved in the regulation of cancer metastasis.

## Introduction

Metastasis is a key step of cancer progression that indicates a more advanced stage and a poorer prognosis. Multiple cellular processes, including the degradation of the extracellular matrix (ECM), the epithelial-to-mesenchymal transition (EMT), tumor angiogenesis, the development of an inflammatory tumor microenvironment, and the dysfunction of programmed cell death machinery, have been demonstrated to be essential for cancer metastasis [1]. Any mistakes made by a metastatic cell during these cellular events may lead to cell death. Therefore, the regulation of cell death is critical for cancer cells to survive during metastasis.

Programmed cell death is defined as regulated cell death mediated by an intracellular program. Apoptosis was originally thought to be the only form of programmed cell death. However, in the last decade, programmed cell death has expanded to include autophagy and a form of necrosis termed necroptosis (programmed necrosis). Programmed cell death, especially apoptosis and necroptosis, are natural barriers that restrict malignant cells from surviving and disseminating. However, cancer cells evolve various strategies to evade programmed cell death by generating genetic mutations or epigenetic modifications in the key modulators of programmed cell death pathways.

In this review, we summarize the interplay (or the link) of the different form of program cell death with cancer metastasis, and we anticipate future challenges and unsolved questions related to these topics.

## Review

### An introduction to cancer metastasis

Cancer metastasis is a complex process that can be divided into five major steps: the first step, invasion, is characterized by increased cell motility caused by alterations in cell-cell and cell-ECM interactions [2]. The second step is intravasation, in which tumor cells escape from the primary site and migrate into circulation systems. The third step, dissemination, is the process in which malignant cells travel through the circulation systems to reach a capillary bed, where the cancer cells adhere to the vessel walls or are detained at these sites because of size constraints. The fourth step is extravasation, in which cancer cells permeate the vessels to enter their destination organs. Colonization is the final step, in which metastatic cells proliferate and form micrometastases or macrometastases [2]. Alternatively, metastasis can be considered as a two-phase process according to a new perspective [3]: the first phase involves the physical translocation of a cancer cell to a distant organ, whereas the second phase encompasses the process of the development of the cancer cells into a metastatic lesion at the distant site. Typically, the initial steps of metastasis (invasion, intravasation, dissemination, and extravasation) proceed at a very high efficiency, but the final step, colonization, is less efficient. It has been estimated that only ~0.01% of circulating tumor cells ultimately produce macrometastases [4]. This inefficiency may be closely related to the activation of cell death machinery by various stresses before or after the cells reach a new environment. Such stresses include the loss of cell-cell contacts, the recognition and destruction of the cancer cells by the immune system, and the lack of necessary growth factors, all of which may trigger programmed cell death, including apoptosis, autophagy and necroptosis [4].

### Apoptosis and cancer metastasis

Apoptosis is a type of programmed cell death that is characterized by cell membrane blebbing, cell shrinkage, nuclear fragmentation, chromatin condensation, and chromosomal DNA fragmentation [5,6]. There are two basic apoptotic signaling pathways: the extrinsic and the intrinsic pathways [7]. The intrinsic apoptotic pathway is activated by various intracellular stimuli, including DNA damage, growth factor deprivation, and oxidative stress. It relies on the formation of a complex termed the apoptosome, composed of procaspase-9, apoptotic protease-activating factor (Apaf-1), and cytochrome c. A series of Bcl-2 family members, such as Bax, Bak, Bcl-2, and Bcl-x_L_, control the release of cytochrome c by regulating mitochondrial membrane permeabilization. The extrinsic pathway of apoptosis is initiated by the binding of death ligands [e.g., Fas ligand (FasL), TNF-related apoptosis inducing ligand (TRAIL), and TNF-α] to death receptors of the TNF receptor superfamily. This interaction is followed by the assembly of the death-inducing signaling complex (DISC), which consists of the Fas-associated death domain (FADD) protein and procaspase-8/10. DISC then either activates downstream effector caspases (caspase-3, 6 and 7) to directly induce cell death or cleaves the Bcl-2 family member Bid into tBid to activate the mitochondria-mediated intrinsic apoptotic pathway [7]. Numerous factors, such as p53, cellular inhibitor of apoptosis proteins (cIAPs), and NF-κB, have been reported to be involved in the regulation of apoptotic pathways [2,8]. Many small molecules targeting apoptotic pathways have been developed for cancer therapy. For example, ABT-737, ABT-263, and GX15-070 have been reported to act on Bcl-2 family members; GDC-0152, birinapant, AT-406, and HGS-1029 have been designed to antagonize cIAPs, among the most promising targets for anti-cancer agent development; and Nutlins, MI-219 and MI-77301 have been shown to antagonize murine double-minute 2 (MDM2), a critical negative regulator of p53 that promotes p53 ubiquitination and degradation [9,10].

Apoptosis may block metastatic dissemination by killing misplaced cells. Thus, apoptosis serves as an important process for inhibiting metastasis. The success of the metastatic process relies on the ability of malignant cells to escape apoptosis. Apoptotic resistance is indispensable for all steps of metastatic progression, but the most critical step may be the resistance to cell death induced by the loss of cell-cell and cell-ECM contacts [8]. The detachment of cells from the ECM induces a type of apoptosis termed anoikis. Numerous reports have demonstrated that anoikis resistance is frequently observed in metastatic cells [11-14]. For instance, TrkB, a neurotrophic tyrosine kinase receptor, was found to act as a specific suppressor of caspase-associated anoikis in non-malignant epithelial cells. TrkB activates the phosphatidylinositol-3-OH kinase (PI3K)/protein kinase B (PKB) pathway to promote the formation of large cellular aggregates that survive and proliferate in suspension, and these cellular aggregates develop into rapidly growing tumors that infiltrate lymphatics and blood vessels to colonize a distant organ in mice [13]. In addition, signal transducer and activator of transcription 3 (STAT3) was found to play a role in conferring anoikis resistance to pancreatic cancer cells and in promoting metastasis. Enhanced STAT3 expression and phosphorylation at Tyr 705 have been associated with the anoikis resistance and the metastatic capacity of pancreatic cells [14].

In addition, metastatic cells must develop a mechanism to evade cell death resulting from recognition and destruction by cytotoxic lymphocytes such as natural killer (NK) cells. Furthermore, tumor cells must survive in the environment of reactive oxygen species (ROS) produced by endothelial cells when crossing the vessel or tissue barrier during the extravasation step [15,16]. Finally, malignant cells must tolerate hypoxic conditions and proliferate in an environment lacking the necessary cytokines for growth to achieve successful colonization at their destination sites [8,17].

Even after the successful formation of micrometastases, macroscopic tumors may not develop because of dormancy [18]. The nature of dormancy remains to be elucidated. One hypothesis states that the rate of proliferation is balanced by the rate of apoptosis such that no net tumor growth occurs [19], whereas another hypothesis states that dormant cells neither proliferate nor undergo apoptosis [20]. However, genetic variation and/or environmental stimulation may drive cells out of dormancy and into an anti-apoptotic, highly proliferative state [21].

In the Table 1, we summarize the roles of the known major apoptotic factors that participate in cancer metastasis.

**Table 1:** **Roles of the major known apoptotic participators in cancer metastasis**

**Gene**	**Description**	**Association with cancer metastasis (representative examples)**
**1. Caspases and caspase inhibitors**		
Caspase-8	Initiator caspase	Caspase-8 knockout Th-MYCN mice developed advanced neuroblastoma with bone marrow metastasis [22].
Caspase-10	Initiator caspase	Caspase-10 mutations were identified in NSCLC patients with lymph node metastases [23].
Caspase-3	Effector caspase	The caspase-3 protein level negatively correlated with lymph node metastasis in NSCLC patients [24]. Another report described an inverse association between caspase-3 expression and lymph node metastasis in gastric carcinomas, although most of the caspase-3 protein was not activated [25].
IAPs (XIAP, survivin, and cIAP1/2)	Caspase inhibitors	Increased levels of the apoptosis inhibitor protein XIAP contributed to the anoikis resistance of circulating human prostate cancer metastatic precursor cells [26]. A recent study showed that intermolecular cooperation between XIAP and survivin stimulated tumor cell invasion and promoted metastasis and that this pathway was independent of the IAP-mediated inhibition of cell death [27].
DAPK	Upstream regulator of capases-3/6/7	DAPK downregulation or inactivation was observed in several metastatic cancers. In certain cases, DAPK downregulation correlated with metastatic recurrence [28].
**2. Intrinsic apoptotic pathway**		
Apaf-1	Key apoptosome component	Apaf-1 gene haploinsufficiency correlated with colorectal carcinoma progression and hepatic metastasis [29].
Bcl-2	Controls mitochondrial membrane permeability	The pulmonary metastatic burden was dramatically augmented in mice inoculated with Bcl-2 transfectants [30]. Elevated nuclear expression of Bcl-2 correlated with increased hepatocellular carcinoma metastasis [31].
Bcl-x_L_	Controls mitochondrial membrane permeability	Bcl-x_L_ overexpression caused apoptosis resistance and acted as an enhancer of metastasis but not primary tumor growth [32].
Bax	Same as above	Bax expression was markedly decreased in metastatic colorectal cancer cells [33]. Bax inhibitor-1 enhanced cancer metastasis [34].
Maspin	Serine protease inhibitor	Maspin expression was reduced in brain-metastasized breast cancer cells [35]. Decreased expression of maspin restricted the growth and metastasis of colorectal cancer xenografts in mice [36].
**3. Extrinsic apoptotic pathway**		
FADD	Key adaptor that transmits death signals mediated by death receptors	Somatic mutations in FADD were observed at a higher frequency in metastatic NSCLC tumors than in the corresponding primary tumors [23]. High FADD expression was associated with regional and distant metastasis in squamous cell carcinoma of the head and neck [37].
FasL and Fas	Key death ligand and its receptor, respectively	Fas-sensitive melanoma clones were highly tumorigenic but were rarely metastatic in wild-type syngeneic mice. However, in FasL-deficient mice, both the incidence and the number of metastases were increased [38]. The ability of osteosarcoma cells to form lung metastases inversely correlated with cell surface Fas expression [39].
sFas and DcR3	soluble Fas and FasL decoy receptor, respectively	In gastric carcinomas, the serum DcR3 levels closely correlated with the tumor differentiation status and the TNM classification [40].
TRAIL	TNF family death ligand	Mice depleted of NK cells or treated with a TRAIL-blocking antibody exhibited a significant increase in spontaneous liver metastasis [41,42].
DR4 and DR5	Death receptors for TRAIL	TRAIL receptor deficiency in mice enhanced lymph node metastasis of squamous cell carcinoma without affecting primary tumor development [43].
DcR1, DcR2, and OPG	TRAIL decoy receptors	The expression of decoy receptors in tumor cells served as an alternate mechanism to resist TRAIL-induced apoptosis [42].
**4. Regulators of apoptotic pathways**		
JNKs	Dual-role regulators of apoptosis	JNKs induced or inhibited cancer cell apoptosis in a manner that was dependent on the cell type, the stimulus, the duration of JNK activation and the activity of other pathways [44]. JNKs served dual roles as both suppressors and promoters of cancer metastasis [45-47].
NF-κB	Transcription factor	Activated NF-κB transactivated many anti-apoptotic genes, including Bcl-2, Bcl-x_L_, survivin, cIAP-1/2, and c-FLIP, as well as many angiogenesis-related genes [48]. NF-κB activity was closely associated with cancer metastasis [49,50].
p53 and p63	Transcription factors	p53 upregulated pro-apoptotic genes, such as Fas, DR5, Bax, Bak and Apaf-1, and repressed anti-apoptotic effectors, such as Bcl-2, Bcl-x_L_ and survivin [51]. p53 loss or mutation promoted tumor metastasis [44]. The loss of p53 led to invasion and lymph node metastasis of carcinogen-induced colorectal tumors [52]. By interacting with mutant p53, p63 suppressed tumorigenesis and metastasis [53,54].
TGF-β, TβRI/II, and SMADs	TGF-β pathway genes	The SMAD complex transactivated a series of apoptosis-related genes [55-58]. TGF-β signals also induced apoptosis via the activation of the ARTS and Daxx-JNK pathways [59,60]. Prior to tumor initiation and the early stages of progression, TGF-β signaling acted as a tumor suppressor; however, at later stages, it often promoted metastasis [61].
MMPs	Prominent family of proteinases	MMPs played roles in the regulation of ECM turnover, cancer cell migration, cell growth, inflammation, and angiogenesis [62]. They also interfered with the induction of apoptosis in malignant cells via the cleavage of ligands or receptors in the apoptotic pathways [63-65].

**Note:** NSCLC, non-small-cell lung cancer; Apaf-1, apoptotic protease-activating factor; IAPs, cellular inhibitors of apoptosis proteins; XIAP, X-linked inhibitor of apoptosis; DAPK, death-associated protein kinase; FADD, Fas-associated death domain-containing protein; sFas, soluble Fas; DcR3, decoy receptor 3; TRAIL, TNF-related apoptosis-inducing ligand; DcR1, decoy receptor 1, also referred to as TRAIL-R3; DcR2, decoy receptor 2, also referred to as TRAIL-R4; OPG, osteoprotegerin; DR4, death receptor 4; TβR I/II, TGF-β receptor I/II; MMPs, matrix metalloproteinases; JNK, c-Jun N-terminal kinases.

Among these modulators of apoptosis in Table 1, the c-Jun N-terminal kinases (JNK), TGF-β, and matrix metalloproteinase (MMP) pathways play dual roles in apoptosis and metastasis. JNKs, a subgroup of the MAP kinase superfamily, are indispensable for both cell proliferation and apoptosis. Whether the activation of JNKs leads to cell proliferation or apoptosis is dependent on the cell type, the nature of the death stimulus, the duration of its activation and the activities of other signaling pathways [66]. In the absence of NF-κB activation, enhanced JNK activation contributes to TNF-α induced apoptosis. JNK is also a critical mediator of UV radiation-induced apoptosis. JNK promotes apoptosis via different mechanisms. Activated JNK translocates to the nucleus and transactivates c-Jun and other transcription factors (e.g., p53), which further transactivate various pro-apoptotic genes, such as Fas-L, Bak, and p53-upregulated modulator of apoptosis (PUMA) [67]. In addition, JNKs contribute to apoptosis by modulating the activities of mitochondrial pro- and antiapoptotic proteins via distinct phosphorylation events. However, JNK inhibits apoptosis in IL-3-dependent hematopoietic cells via the phosphorylation and antagonism of the proapoptotic Bcl-2 family protein BAD [66].

It was reported that JNK signaling prevented the progression of invasive adenocarcinoma in PTEN^−/−^ prostate cancer. Mice exhibiting JNK deficiency in the prostate epithelium (JNK and PTEN double-deficient mice) develop androgen-independent metastatic prostate cancer more rapidly than control (PTEN-deficient) mice. In addition, JNK-deficient progenitor cells exhibited increased proliferation and tumorigenic capacity compared with progenitor cells from control prostate tumors [45].

A group reported that Notch and myocyte enhancer factor 2 (Mef2) cooperated to promote proliferation and metastasis via JNK signal activation and the consequent induction of the invasion marker MMP1 in a Drosophila model [46]. Another study showed that receptor for advanced glycation end products (RAGE) splice variant 1 inhibited tumor formation, cell invasion, and angiogenesis induced by RAGE ligand signaling, which was closely related to the strong suppression of JNK by this splice variant protein [47].

The transforming growth factor-β (TGF-β) family proteins bind to cell surface type I and type II serine/threonine kinase receptors (TGFβRI and TGFβRII, respectively) and SMAD mediators to regulate many biological processes. Numerous studies have reported roles of the TGF-β pathway in apoptosis. Many pro-apoptotic genes are under the control of the SMAD transcription-regulating complexes. For example, TGF-β-inducible early response gene (TIEG1) [56], death-associated protein kinase (DAPK) [57], and SH2 domain-containing inositol-5-phosphatase (SHIP) [58] have been shown to be essential for the suppression of cell proliferation and the induction of apoptosis in many cell types. Another mechanism of TGF-β-related apoptosis is the induction of the mislocalization of a mitochondrial septin family member ARTS. Upon translocation, ARTS binds to and inactivates XIAP, a key inhibitor of apoptosis, which leads to the activation of caspase-3 and apoptosis [59]. In addition, TGF-β signals induce the death associated protein (Daxx)-JNK pathway to induce apoptosis under certain circumstances [60].

The roles of the TGF-β signaling pathway in cancer development are uncertain [61]. Prior to tumor initiation and during the early stage of progression, TGF-β acts as a tumor suppressor via cell cycle arrest and the induction of apoptosis; however, at advanced stages, TGF-β often acts as a tumor promoter via the acceleration of the EMT, invasion, and angiogenesis, the maintenance of tumor stem cells, and the alteration of the tumor microenvironment [61]. Interestingly, TGF-β is expressed at high levels during the late stages of tumor progression in many human cancers, but paradoxically, the TGF-β pathway is frequently mutationally inactivated in cancer cells. Further studies revealed that elevated TGF-β expression played an important role in promoting stromal cells to secret cytokines that favor cancer cell metastasis [68].

Matrix metalloproteinases (MMPs) constitute one of the most prominent families of proteinases associated with tumor metastasis. MMPs interfere with the induction of apoptosis in malignant cells via the cleavage of ligands or receptors in apoptotic pathways [62]. For instance, MMP-7 was reported to cleave membrane-bound FasL on doxorubicin-treated cancer cells, thereby attenuating apoptosis and increasing the resistance of these cells to chemotherapy [63,64]. MMP-13 was shown to be involved in the shedding of nerve/glial antigen 2 (NG2), a novel anoikis receptor, thereby contributing to the attenuation of anoikis [65].

### Autophagy and cancer metastasis

Autophagy is an evolutionarily conserved catabolic process in which intracellular membrane structures package protein complexes and organelles to degrade and renew these cytoplasmic components. It is thus critical for cell growth regulation and internal homeostasis [69]. Autophagy is physiologically a cellular strategy and mechanism for survival under stress conditions. When over-activated under certain circumstances, excess autophagy results in cell death. To date, three types of autophagy have been identified: macroautophagy, microautophagy, and chaperone-mediated autophagy. We only discuss macroautophagy in this review; therefore, henceforth, "autophagy" specifically refers to macroautophagy. Autophagy is a multi-step process that includes nucleation, elongation, and autophagosome and autolysosome formation and that is executed by a series of highly conserved genes termed autophagy-related genes (ATGs) [70]. Autophagy is often triggered by nutrient deprivation, ROS, hypoxia, drug stimuli, and endoplasmic reticulum (ER) stress via complex signal transduction pathways. Alterations in the autophagy machinery may lead to diverse pathological conditions, such as neurodegeneration, ageing, and cancer [71]. Mammalian target of rapamycin complex 1 (mTORC1), class I PI3K, AKT, class III PI3K, Beclin-1 and p53 are critical components of the autophagic pathway that have become major targets of autophagy-related drug design. Numerous small molecules have been found to target these components and to play a role in tumor treatment. For example, rapamycin and its derivatives (i.e., rottlerin, PP242 and AZD8055) target the PI3K/AKT/mTOR signaling pathway to induce autophagy; spautin-1 and tamoxifen regulate Beclin-1 activity to inhibit and promote autophagy, respectively; and oridonin and metformin trigger p53-mediated autophagy and cell death [72].

The role of autophagy in cancer metastasis is complex, as reports have indicated both pro-metastatic and anti-metastatic roles of autophagy. Stage-specificity may affect the cellular response to autophagy during cancer metastasis [73]. During the early stage of cancer metastasis, autophagy may act as a suppressor of metastasis by restricting tumor necrosis and inflammatory cell infiltration and by alleviating oncogene-induced senescence. These processes may help to reduce the invasion and dissemination of cancer cells from the primary site. During the advanced stages of metastasis, autophagy tends to act as a promoter of metastasis by promoting ECM-detached metastatic cell survival and colonization in a distant site and by inducing metastatic cells that fail to establish contact with the ECM in the new environment to enter dormancy.

#### The anti-metastatic role of autophagy

Necrosis frequently occurs inside a tumor due to hypoxia and metabolic stress, which enables inflammatory cells, especially macrophages, to infiltrate tumor sites and which generates a favorable microenvironment for tumor metastasis [74,75]. Autophagy facilitates the survival of tumor cells under metabolic stress and hypoxic conditions, thereby effectively reducing tumor necrosis and subsequent immune cell infiltration and metastasis [76]. In addition, autophagy regulates the selective release of the immune modulator high-mobility group B1 (HMGB1) by the tumor cells that are destined to die [77,78]. Once released, HMGB1 activates dendritic cells by engaging Toll-like receptor 4, which triggers an intense antitumor immune response and restricts metastasis [79,80]. Prophylactic treatment with the TLR4 and TLR9 agonist complex triggered anti-metastatic immunity and impaired tumor metastasis by inducing the autophagy-associated death of melanoma cells via IFN-γ/STAT1 activation. The induction of autophagy via the injection of rapamycin with or without the TLR4/9 agonist complex into the tumor attenuated metastasis [81].

ATG5, a key regulator of autophagy, was found to be downregulated in primary melanomas compared to benign nevi, and this decrease in ATG5 expression is accompanied by a reduction in the expression of LC3 and in basal autophagy. It was shown that patients expressing low levels of ATG5 in their tumors exhibited decreased progression-free survival according to a follow-up of 158 primary melanoma patients. Mechanically, reducing ATG5 expression may promote cell proliferation by preventing oncogene-induced senescence and may contribute to the progression of early-stage cutaneous melanoma [82].

The PI3K/Akt/mTOR pathway is a critical signaling pathway that negatively regulates autophagy and that promotes cancer progression. Recent studies have suggested that PI3K/Akt/mTOR signaling is upregulated in 30-50% of prostate cancers, often due to the loss of PTEN. It has been reported that molecular changes in the PI3K/Akt/mTOR signaling pathway are implicated in the elevation of the tumor stage and grade and the risk of recurrence [83]. PTEN mutations and deletion within primary tumors have been associated with an increased risk of metastasis, and early targeting of PTEN may prevent metastasis [84]. Genistein, an Akt inhibitor, has been shown to play a role in decreasing the incidence of lung metastasis in an orthotopic prostate model using PC-3 cells [85]. Mechanically, increasing autophagic flux by inhibiting the PI3K/Akt/mTOR pathway may promote the apoptosis of cancer cells [86,87].

In addition, a unique death modality termed autophagic cell death plays a role in impeding cancer metastasis. Autophagic cell death refers to cell death caused by autophagy rather than cell death with autophagy. Thus, the ultimate cell death process of autophagic cell death is executed by over-activated autophagic flux rather than apoptosis or necroptosis. The genetic or drug-based inhibitors of autophagy, but not apoptosis or necroptosis inhibitors, rescue this type of cell death [88,89]. It is known that there is a complex relationship between apoptosis and autophagy. Typically, autophagy antagonizes apoptosis, and as a feedback response, apoptosis-related caspase activation reduces the autophagic process. However, autophagy can also trigger apoptosis under certain circumstances via the activation of caspase-8 and the depletion of endogenous apoptosis inhibitors [90-92]. Autophagic cell death does not include autophagy-induced apoptosis or necroptosis. Several studies have suggested a potential relationship between autophagic cell death and cancer metastasis. For example, one study showed that blocking the CXCR4/mTOR signalling pathway induced autophagic cell death and the anti-metastatic properties of peritoneally disseminated gastric cancer cells [93].

#### The pro-metastatic role of autophagy

Acquiring the ability to survive and proliferate in the absence of the ECM while disseminating through the circulation systems and colonizing a distant site is necessary for cancer cell metastasis [94,95]. Otherwise, cancer cells die of anoikis (a specific type of apoptosis induced by the loss of ECM attachment). The constitutive activation of pro-survival signals such as PI3K, Ras–ERK, NF-κB, and Rho GTPase often occurs in cancer cells, thereby antagonizing anoikis. This antagonism can be achieved via the autocrine secretion of growth factors or the overexpression of receptor tyrosine kinases [13,96]. Accumulating evidence suggests that autophagy also provides a mechanism for matrix-detached pre-metastatic tumor cells to avoid anoikis [97,98]. In a hepatocellular carcinoma (HCC) lung metastasis model, the inhibition of autophagy (via the lentivirus-mediated silencing of BECN1 and ATG5) markedly decreased the pulmonary metastasis of HCC cells. Further investigation indicated that the inhibition of autophagy did not affect cell invasiveness, migration or the EMT but attenuated the anoikis resistance and lung colonization of HCC cells [99]. Another study showed that autophagy was induced by either matrix detachment or β1 integrin inhibition [100]. Autophagy in ECM-disrupted cells may compensate for the loss of extrinsic signals that promote and maintain nutrient and energy metabolism. Under these conditions, autophagy might delay the onset of apoptosis, providing cells with additional time to re-attach to an appropriate ECM. In a rapidly growing tumor with high energy and biosynthesis requirements, detachment-induced autophagy undoubtedly increases the survival of the cells deprived of ECM contact [96,97].

Aside from ECM disruption, increased metabolic and oxidative stresses in cancer cells and adverse environmental stresses are major barriers to the metastasis of cancer cells. Autophagy-defective KRAS-driven lung cancer cells exhibited impaired mitochondrial energy homoeostasis, oxidative stress and a constitutively active DNA damage response that were further mediated by p53 and that triggered apoptosis in malignant cells, suggesting that autophagy may play an important role in the maintenance of mitochondrial function and in the clearance of unfavorable factors the induce cell death, thus promoting tumor progression [101,102]. Autophagy may also promote the survival of HCC under hypoxic conditions via the activation of mitochondrial β-oxidation and intracellular ATP production [103].

Disseminated tumor cells that are unable to form firm ECM contacts in a foreign microenvironment may transform to enter dormancy [104], which may allow the tumor cells to survive for years or decades at distant sites without developing into secondary tumors while retaining the ability to metastasize under the appropriate conditions. Lu et al. reported that the tumor suppressor aplasia Ras homolog member I (ARHI) induced autophagy and enhanced the survival of dormant tumor cells in vivo, demonstrating an association between autophagy and the regulation of cancer cell dormancy for the first time [105]. Therefore, it is possible that the partially disseminated tumor cells that cannot successfully establish an interaction with the ECM may initiate autophagy, which drives the tumor cells into dormancy and promotes their survival.

**Figure 1:**
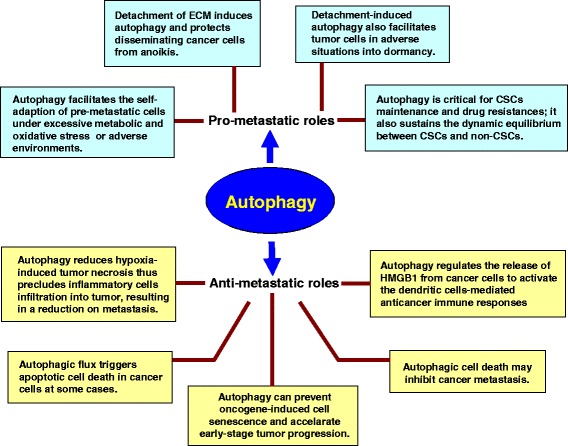
**Contradictory effects of autophagy on cancer metastasis.** The text in the wathet boxes summarizes the possible pro-metastatic mechanisms of autophagy, and the text in the yellow boxes depicts the potential anti-metastatic mechanisms of autophagy. HMGB1, high-mobility group B1; TLR4, Toll-like receptor 4; CSCs, cancer stem cells.

Cancer stem cells (CSCs) are characteristically resistant to conventional anticancer therapy, which may contribute to treatment failure and tumor relapse. CSCs exhibit the potential to regenerate for an indefinite period, which may promote tumor metastasis [106]. Recently, autophagy has been shown to be a critical factor for CSC survival and drug resistance [107,108]. Malignant breast tissue contains a rare population of multi-potent cells exhibiting the capacity to self-renew; these cells are defined as CSCs. These mammary CSCs can propagate in culture as floating spherical colonies termed ‘mammospheres’. The key autophagy protein Beclin 1 was more strongly expressed in mammospheres established from human breast cancer samples or cell lines than in the parental adherent cells, resulting in a higher level of autophagy in mammospheres. This prosurvival autophagic flux was important for CSC maintenance and tumor progression [108]. In addition, one group reported that HIF-1a and autophagy played a role in modulating the conversion of non-stem pancreatic cancer cells to stem cells. This result suggested a role of HIF-1a and autophagy in sustaining the dynamic equilibrium between CSCs and non-CSCs [109]. The relationships between autophagy and cancer metastasis are depicted in Figure 1.

### Necroptosis and metastasis

Necrosis was originally considered to be an accidental and unregulated cell death. Accumulating evidence has shown that necrosis can be induced and proceed in a regular manner like apoptosis, although in a caspase-independent fashion. Regulated necrosis is termed “programmed necrosis” or “necroptosis” to distinguish it from necrosis caused by physical trauma [110]. Necroptosis can be induced by the activation of the TNF receptor superfamily [111], T cell receptors [112], interferon receptors [113], Toll-like receptors (TLRs) [114], cellular metabolic and genotoxic stresses, or various anti-cancer agents. It can be pharmacologically inhibited by chemical compounds such as necrostatin-1 (Nec-1) [115]. The formation of the “necrosome” by receptor-interacting protein kinase 1 (RIP1) and RIP3 is one of the most critical characteristics of necroptosis. It is a multi-step process that contains three key checkpoints. For example, in TNF-α mediated necroptosis [110], at the first checkpoint, the E3 ligases cellular inhibitor of apoptosis 1 (cIAP1) and cIAP2 induce RIP1 ubiquitination [116], which blocks necroptosis via NF-κB-dependent or -independent mechanisms. The removal of ubiquitin chains from RIP1 by the deubiquitinase cylindromatosis (CYLD) is critical for the packaging of Complex IIa (including caspase-8, FADD, and RIP1) and Complex IIb [(including caspase-8, FADD, RIP1, RIP3, and mixed lineage kinase domain-like (MLKL)] [117]. At the second checkpoint, activated caspase-8 cleaves and abolishes the activities of RIP1, RIP3, and CYLD [118-120]. Cleaved RIP1 and RIP3 lose their capabilities of trans-phosphorylation and downstream substrate phosphorylation. At the third checkpoint, when the disruption of RIP1 and RIP3 is prevented by caspase-8 inhibitors (i.e., zVAD) or by the genetic inhibition of caspase-8 or FADD, the trans-phosphorylation of RIP1 and RIP3 promotes their aggregation into the filamentous-like necrosome [110]. MLKL is further phosphorylated by RIP3 and is recruited to the necrosome by its interaction with RIP3 [121]. MLKL forms a homotrimer via its amino-terminal coiled-coil domain and translocates to the plasma membrane during TNF-induced necroptosis, which leads to necrotic plasma membrane permeabilization [122]. In addition to MLKL, phosphoglycerate mutase 5 (PGAM5) is a downstream substrate of RIP3 [123].

Several molecules and pathways contribute to the execution of TNF receptor-mediated necroptosis. Some of these effectors are also involved in other receptor-mediated necroptosis pathways [124]. During the progression of necroptosis, ROS are generated [125,126], resulting in lipid peroxidation and increased mitochondrial membrane permeability. The cytosolic ATP levels are sharply reduced due to the attenuation of ATP transport from the mitochondria to the cytosol and ATP consumption by over-active poly(ADP-ribose) polymerase 1 (PARP1). Apoptosis-inducing factor (AIF) is released from mitochondria due to the increase in the mitochondrial membrane permeability and enters the nucleus to cleave DNA. Lysosomal membrane permeabilization (LMP) also occurs during necroptosis, resulting in the leakage of cytotoxic hydrolases into the cytosol [124]. In addition, dynamin-related protein l (Drp1), which acts downstream of PGAM5, is thought to regulate mitochondrial fission to execute necroptosis [123].

Necroptosis plays an indispensable role during normal development. Moreover, it has been implicated in the pathogenesis of a variety of human diseases, including cancer [127]. The necroptosis machinery is often impaired during tumorigenesis and tumor progression. For example, chronic lymphocytic leukemia (CLL) cells failed to undergo necroptosis upon stimulation using TNFα combined with the pan-caspase inhibitor zVAD. Two key components of necroptotic machinery, RIP3 and CYLD, were markedly downregulated in CLL [128]. In non-Hodgkin lymphoma, single nucleotide polymorphisms (SNPs) in the RIP3 gene were detected in 458 patients and correlated with increased risk of non-Hodgkin lymphoma, which indicates that genetic variations in the RIP3 gene may contribute to the onset of this disease [129]. There is a growing list of compounds and anticancer agents that have been shown to induce necroptosis in cancer cells. Shikonin was the first reported small molecule that induced necroptosis [130]. Numerous studies have shown that shikonin and its analogs not only are highly tumoricidal but also exhibit the ability to bypass drug resistance machineries [130-133]. In addition, compounds such as 5-benzylglycinyl-amiloride, obatoclax, and D-galactose employ necroptosis to kill malignant cells [134-136]. Notably, some traditionally pro-apoptotic anti-cancer agents have recently been demonstrated to share the ability to induce the necroptosis of tumor cells under certain circumstances. For example, interferon-β-armed oncolytic adenovirus (ZD55-IFN-β) induced both apoptosis and necroptosis in cancer cells. However, Nec-1 treatment converted ZD55-IFN-β-induced necroptosis to apoptosis [137]. In addition, although TRAIL is a well-known apoptosis inducer, one study showed that acidic extracellular pH converted TRAIL-induced apoptosis to necroptosis in human HT29 colon and HepG2 liver cancer cells via a process that involved RIPK1/RIPK3-dependent PARP-1 activation [138].

To date, few studies have associated necroptosis with metastasis. Fu et al. reported that shikonin greatly reduced the lung metastasis of osteosarcoma by inducing RIP1- and RIP3-dependent necroptosis [132]. The induction of a high level of ROS via necroptosis may represent one factor that restricts cancer cell metastasis [139]. As we have described above, metastatic cells in the circulation or at new sites must survive in an environment without interacting with the ECM; under these conditions, tumor cells face major difficulties in maintaining nutrient and energy equilibration and in antagonizing metabolic stresses, especially ROS. Disseminated tumor cells have evolved different strategies to restore their ATP levels and to restrict cellular ROS production, including the over-activation of pro-survival signals (such as PI3K, Ras-ERK, and NF-κB), the enhancement of antioxidant activity, the altered activation of metabolic pathways (preferentially the glycolysis and pentose phosphate pathways), and the initiation of autophagy. However, necroptosis represents another mechanism to eliminate metastatic cancer cells by triggering ROS bursts. RIP3 has been observed to be critical for regulating ROS production during necroptosis [126], and this finding is in agreement with another study showing that RIP3 activates several metabolic enzymes [including glycogen phosphorylase (PYGL), glutamate-ammonia ligase (GLUL), and glutamate dehydrogenase 1 (GLUD1)] to regulate TNF-induced ROS production. Silencing each of these enzymes results in decreased levels of ROS accumulation and cell death [125]. Therefore, it appears to be reasonable that necroptosis is an important mechanism that restricts tumor metastasis. In this case, tumor cells must overcome both anoikis and necroptosis to successfully metastasize.

### Interaction between apoptosis, autophagy, necroptosis, and cancer metastasis

**Figure 2:**
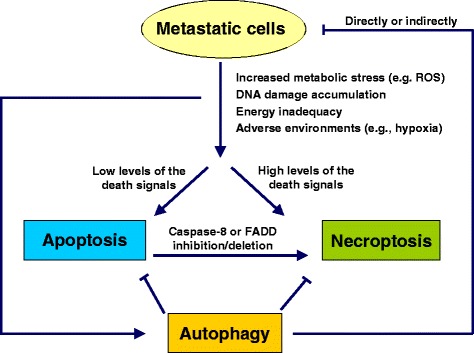
**Interaction between different types of programmed cell death and cancer metastasis.** Disseminating metastatic cells must face many unfavorable conditions, including detachment from the ECM, attack by immune cells, hypoxia or a growth factor-lacking environment, that cause increased cellular ROS production and DNA damage and insufficient energy status. Low levels of death signals stimulate apoptosis, whereas high levels of death signals often result in necroptosis. Due to the activity of the apoptosis (anoikis) and necroptosis machineries, most metastatic cells from the primary tumor cannot successfully macrometastasize. Compared with apoptosis and necroptosis, autophagy appears to be fairly capricious, as on one hand, autophagy greatly improves the fitness of metastatic cells under stressful conditions to counteract apoptosis and necroptosis, but on the other hand, autophagy reduces metastasis by restricting tumor necrosis and by precluding inflammatory immune cell infiltration. Additionally, excess autophagy induces the death of metastasizing cells.

Programmed cell death in vivo involves the complex interaction between apoptosis, autophagy, and necroptosis [140]. In some cases, a specific stimulus triggers only one type of programmed cell death, but in other situations, the same stimulus may initiate multiple cell death processes. Different types of mechanisms may co-exist and interact with each other within a cell, but ultimately, one mechanism dominates the others. The decision taken by a cell to undergo apoptosis, autophagy, or necroptosis is regulated by various factors, including the energy/ATP levels, the extent of damage or stress, and the presence of inhibitors of specific pathways (e.g., caspase inhibitors). ATP depletion activates autophagy. However, if autophagy fails to maintain the energy levels, necroptosis occurs [141]. Slight/moderate damage and low levels of death signaling typically induce apoptosis, whereas severe damage and high levels of the death signaling often result in necroptosis [142]. Although apoptosis is often the first mode of cell death and although necroptosis is triggered only as a backup mechanism to ensure that cell death occurs, emerging evidence has shown that the necroptotic pathway may predominate under certain pathological conditions [142]. The complex relationships between different types of cell death and cancer metastasis are depicted in Figure 2.

In the course of cancer metastasis, malignant cells must overcome a series of unfavorable conditions, including detachment from the ECM, attack by immune cells, hypoxia and a growth factor-lacking environment, which cause increased cellular ROS production and DNA damage and an insufficient energy status. Therefore, most metastatic cells from the primary tumor are unable to successfully macrometastasize and are killed via apoptosis or necroptosis. On one hand, autophagy greatly improves the fitness of cancer cells under stressful conditions and, thus, attenuates apoptosis and necroptosis, but on the other hand, autophagy antagonizes metastasis by restricting tumor necrosis and subsequent immune cell infiltration. Additionally, excess autophagy induces the death of metastasizing cells. Therefore, the interaction between different types of cell death and cancer metastasis is highly complex. In addition, cancer cells have evolved sophisticated mechanisms to antagonize apoptosis and necroptosis. However, defects in the machinery of one type of cell death may not affect that of another. Thus, triggering a single type of programmed cell death may not be sufficient for the treatment of cancer metastasis. The selection of different cell death inducers or the combined use of different cell death pathway inducers will help to overcome drug resistance to kill metastatic cells [140,143,144].

## Conclusions and perspectives

Programmed cell death, are natural barriers that restrict malignant cells from surviving and disseminating. However, cancer cells evolve various strategies to evade programmed cell death by generating genetic mutations or epigenetic modifications in the key modulators of programmed cell death pathways. The role of autophagy in cancer metastasis is complex, as reports have indicated both pro-metastatic and anti-metastatic roles of autophagy. Stage-specificity may affect the cellular response to autophagy during cancer metastasis. Programmed cell death in vivo involves the complex interaction between apoptosis, autophagy, and necroptosis. Different types of mechanisms may co-exist and interact with each other within a cell. The decision taken by a cell to undergo apoptosis, autophagy, or necroptosis is regulated by various factors, including the energy/ATP levels, the extent of damage or stress, and the presence of inhibitors of specific pathways.

In this review, we summarized how apoptosis, autophagy, and necroptosis affect cancer metastasis based on the current literature. However, still many issues remain to be clarified.

(i) As we have discussed in this review, autophagy plays a dual role in tumor metastasis. Generally speaking, autophagy may exert an inhibitory effect during the early step of cancer metastasis by, for example, restricting necrosis and inflammation and by preventing oncogene-induced senescence, thereby limiting the invasion and dissemination of cancer cells from the primary site. Alternatively, autophagy tends to promote metastasis during advanced cancer stages by supporting ECM-detached metastatic cell survival and colonization at a distant site and by inducing metastatic cells to enter dormancy if they fail to establish a contact with the ECM in the new environment. As a delicate process, autophagy may play either pro- or anti-metastatic roles depending on the context. At present, the complex role of autophagy in metastasis remains unclear. It is necessary to determine how the dual role of autophagy in metastasis is regulated; i.e., what are the signals, molecules, and mechanisms that enable autophagy to play a dominant anti-metastatic role in one situation an opposite role in another situation.

(ii) At present, we know very little about the roles of necroptosis in cancer progression. As we have mentioned above, the necroptosis machinery may be impaired during tumorigenesis and tumor progression. For example, CLL leukemia cells failed to undergo necroptosis due to the downregulation of RIP3 and CYLD [128]. In non-Hodgkin lymphoma, genetic variations in the RIP3 gene were detected in 458 patients and correlated with increased risk of non-Hodgkin lymphoma [129]. However, according to our study (unpublished) using a series of cancer cell lines, only a small proportion (approximately 10%) of cancer cells undergo necroptosis in response to stimuli. It is necessary to determine why so many cancer cells lose their necroptotic machinery and the importance of this machinery in regulating tumorigenesis and cancer metastasis.

(iii) It is necessary to investigate the relationship between programmed cell death and cancer metastasis in the tumor microenvironment, including the tumor-infiltrating immune cells. For example, preclinical evidence has shown that chemotherapy-induced autophagy in cancer cells may contribute to the recruitment of myeloid cells to the tumors and the subsequent T lymphocyte-mediated suppression of tumor growth [145]. In addition, programmed cell death plays critical roles in the maintenance of proper innate and adaptive immune function [146]. Therefore, dysfunction of the programmed cell death machinery in the immune system may change its effect on cancer growth and metastasis. This evidence suggests that programmed cell death should be investigated simultaneously in both cancer cells and immune cells to understand the interaction between these cell types.

(iv) The regulation of programmed cell death and metastasis by non-coding RNAs (ncRNAs) is a novel direction of this research field. ncRNAs include multiple classes of RNA transcripts that are not translated into proteins but can regulate the transcription, stability or translation of protein-coding genes in the mammalian genome [147]. The most studied ncRNAs are microRNAs (typically consisting of 19–24 nucleotides), which are highly conserved small ncRNA molecules that function to regulate a wide variety of cellular processes by interfering with protein expression or mRNA degradation. Numerous miRNAs have been reported to be involved in the regulation of programmed cell death or cancer progression [148,149]. More recently, long none-coding RNAs (lncRNAs) have been found to play critical roles in transcriptional and translational regulation [150] and have been implicated in a wide range of human diseases, including cancer [151]. Some lncRNAs have been observed to target the apoptotic and autophagy pathways and to play a role in cancer development. For example, MEG3 inhibited the proliferation and induced the apoptosis of NSCLC cells by affecting p53 expression [152], and GAS5 regulated the apoptosis of NSCLC cells [153]. In addition, HULC was overexpressed in human gastric cancer (GC) cell lines and tissues compared with normal controls, and this overexpression correlated with lymph node metastasis, distant metastasis and advanced tumor node metastasis stage. Further investigation showed that HULC-induced autophagy was a major reason for GC cell survival and metastasis [154]. At present, the regulation of programmed cell death and cancer metastasis by ncRNAs, especially lncRNAs, remains largely unknown, and further investigation is required to clarify their mechanisms.

The more we understand the specific roles, mechanisms, and regulators of apoptosis, autophagy, and necroptosis and their interaction with cancer metastasis, the better therapeutic strategies can be developed for cancer treatment.

## Abbreviations

ECM: Extracellular matrix
EMT: Epithelial-to-mesenchymal transition
Apaf-1: Apoptotic protease-activating factor
FasL: Fas ligand
TRAIL: TNF-related apoptosis inducing ligand
DISC: Death-inducing signaling complex
FADD: Fas-associated death domain
cIAPs: Cellular inhibitor of apoptosis proteins
MDM2: Murine double-minute 2
PI3K: Phosphatidylinositol-3-OH kinase
PKB: Protein kinase B
STAT3: Signal transducer and activator of transcription 3
NK: Natural killer
ROS: Reactive oxygen species
NSCLC: Non-small-cell lung cancer
XIAP: X-linked inhibitor of apoptosis
DAPK: Death-associated protein kinase
FADD: Fas-associated death domain-containing protein
sFas: soluble Fas
DcR3: Decoy receptor 3
DcR: Decoy receptor
OPG: Osteoprotegerin
DR4: Death receptor 4
TβR I/II: TGF-β receptor I/II
MMPs: Matrix metalloproteinases
JNK: c-Jun N-terminal kinases
PUMA: p53-upregulated modulator of apoptosis
Mef2: Myocyte enhancer factor 2
RAGE: Receptor for advanced glycation end products
TGF-β: Transforming growth factor-β
TIEG1: TGF-β-inducible early response gene
SHIP: SH2 domain-containing inositol-5-phosphatase
Daxx: Death associated protein
NG2: Nerve/glial antigen 2
ATGs: Autophagy-related genes
ER: Endoplasmic reticulum
mTORC1: Mammalian target of rapamycin complex 1
HMGB1: High-mobility group B1
HCC: Hepatocellular carcinoma
ARHI: Aplasia Ras homolog member I
CSCs: Cancer stem cells
TLRs: Toll-like receptors
Nec-1: Necrostatin-1
RIP1: Receptor-interacting protein kinase 1
CYLD: Deubiquitinase cylindromatosis
MLKL: Mixed lineage kinase domain-like
PGAM5: Phosphoglycerate mutase 5
PARP1: Poly(ADP-ribose) polymerase 1
LMP: Lysosomal membrane permeabilization
Drp1: Dynamin-related protein l
AIF: Apoptosis-inducing factor
CLL: Chronic lymphocytic leukemia
SNPs: Single nucleotide polymorphisms
PYGL: Glycogen phosphorylase
GLUL: Glutamate-ammonia ligase
GLUD1: Glutamate dehydrogenase 1
GC: Gastric cancer
ncRNAs: Non-coding RNAs
lncRNAs: Long none-coding RNAs
